# The use of stunting as a nutrition indicator in Yemen civil war

**DOI:** 10.1186/s12992-019-0502-x

**Published:** 2019-11-08

**Authors:** Nima Yaghmaei, Debarati Guha-Sapir

**Affiliations:** Centre for Research on the Epidemiology of Disasters, Institute of Health & Society, UCLouvain, Brussels, Belgium

**Keywords:** War, Conflict, Malnutrition, Child health, Anthropometry

Humanitarian crises, such as conflicts and disasters, have devastating impacts on health systems, quickly rendering pre-crisis health data outdated. Without comprehensive assessments, response planning and resource allocation become difficult and can result in ineffective actions. We thank El Bcheraoui and colleagues for their insightful research paper ‘Health in Yemen: losing ground in war time’. The paper provides an analysis into the effects of the war in Yemen on public health, and it will assist planning and resource allocation going forward.

As highlighted by El Bcheraoui and colleagues, humanitarian crises have a large impact on maternal and child health, with malnutrition being a major concern. Therefore, one effective method of health assessment in crises is the monitoring of nutrition status in these vulnerable populations. Commonly used indicators for nutrition status are anthropometric indices, such as weight-for-height (WH)(wasting), height-for-age (HA)(stunting), weight-for-age (WA)(underweight), or mid-upper arm circumference (MUAC) measurements. These indices are used by national surveys, such as the Demographic and Health Surveys (DHS) and UNICEF’s Multiple Cluster Surveys (MICS), or small-scale survey methods, such as the Standardized Monitoring and Assessment of Relief and Transitions (SMART).

In the case of Yemen, rates of malnutrition were demonstrated by El Bcheraoui and colleagues using various indicators of nutritional status for both women 15–49 years old and children < 5 years old [1]. However, we would like to bring into question the use of the terminology “global acute malnutrition (GAM) stunting” and “severe acute malnutrition (SAM) stunting” in the results.

The term “Global acute malnutrition (GAM) stunting” is the incorrect combination of two indicators used for malnutrition: “Global acute malnutrition (GAM)” and “stunting.” Stunting is the terminology used for children < 5 years old who have a HA Z-score (HAZ) < 2.0, and is recognized as chronic malnutrition for the indication of linear growth retardation [2, 3]. On the other hand, global acute malnutrition (GAM) is the terminology used for the combination of severe acute malnutrition (SAM) and moderate acute malnutrition (MAM): WH Z-score (WHZ) < 2.0 (wasting) and/or MUAC < 12.5 cm and/or nutritional oedema [4]. Concurrently, the term “SAM stunting” is incorrect as SAM is indicated by WHZ < 3.0 (severe wasting) and/or MUAC < 11.5 cm and/or nutritional oedema, while severe stunting is indicated by HA Z-score < 3.0 [4]. Despite the evidence of some common causality and positive association between stunting and wasting, they remain separate indicators since they are based on different anthropometric measures [2].

The misuse of terminology may have operational implications since GAM prevalence above 15% is considered critical and regarded as the threshold for an emergency, while this is not the case for stunting, since a stunting prevalence of 15% is considered “low” [5, 6]. Thus, the incorrect labelling of stunting as wasting would likely result in the overestimation of malnutrition rates, while the incorrect labelling of wasting as stunting would likely underestimate malnutrition rates. El Bcheraoui and colleagues indicate a national average of 52.3% for “Global acute malnutrition GAM stunting”, this figure is not indicative of GAM which can be expected to be significantly lower, but it is indicative of stunting, which was at 46.5% prior to the conflict [1]. Additionally, data from more recent small-scale surveys conducted in Yemen by humanitarian organizations have also found similar levels of stunting.

Even with the adjusted labelling, the use of stunting as an indicator for malnutrition in a humanitarian crisis also has operational implications. Firstly, fluctuations in stunting and acute malnutrition are not synonymous, and it is not recommend to use stunting as an independent indicator for nutrition interventions in humanitarian crises [7]. Acute malnutrition is a more appropriate indicator as it rapidly manifests in young children and has a higher risk of mortality than chronic malnutrition, while chronic malnutrition is not indicative of an acute crisis as it reflects long-term conditions [7, 8]. Prior to the war, Yemen was ranked poorly on the Human Development Index, and large proportions of the population faced poverty and nutrition deficiencies [1]. Thus, stunting rates from surveys conducted in 2016 would likely be more indicative of pre-crisis conditions, ultimately giving an inadequate indication of the areas with the highest needs as a result of the crisis. As well, acute malnutrition is easier to measure using either WHZ or MUAC, neither of which are overly susceptible to reporting bias for age, unlike stunting (HAZ). Additionally, acute malnutrition indicators can be used as an individual-level indicator for treatment and as a population-level indicator, while stunting is only appropriate at a population-level [7].

From the perspective of intervention design, stunting does not indicate the nutrition information needed for acute nutrition interventions, since, comparatively, stunting indicates to a lesser degree the nutrition status of a population, and more the general living and welfare of the population [9]. Nutrition interventions in humanitarian crises usually focus on immediate needs and are nutrition specific. These interventions may include infant and young child feeding (IYCF) programs, and distribution of food aid and Ready-To-Use Foods (RUFs). For these interventions, stunting would be an inappropriate indicator since success in these interventions would not necessarily correspond with rapid improvements of linear growth amongst the population [7]. As a result, potentially lifesaving short-term interventions could be deemed unsuccessful having had only minor changes in linear growth retardation and thus, only minor changes in stunting rates.

Therefore, of the potential nutrition indicators, reporting using indicators such as WHZ, MUAC and underweight (WAZ) is recommended. If possible, both chronic and acute malnutrition should be measured, since concurrent stunting and wasting exhibit the highest risk of mortality [5].

As highlighted by El Bcheraoui and colleagues, data sources are limited during humanitarian crises. DHS and MICS surveys are difficult to conduct in real time and are time consuming. This further demonstrates the importance and necessity of small-scale surveys. With improvements in statistical analysis using modelling, researchers can utilize small-scale surveys and report crucial information, such as acute malnutrition levels in the population. El Bcheraoui and colleagues have made this clear in their discussion by recommending rapid health assessment surveys for short-term activities. We believe this is an important recommendation that needs more attention.

The paper by El Bcheraoui and colleagues is a reminder of the challenges faced by health systems in humanitarian crises, and the difficulty of producing comprehensive assessments in such circumstances.

## Data Availability

Not applicable

## Abbreviations

DHS: Demographic and Health Survey
GAM: Global Acute Malnutrition
HA: Height-for-age
HAZ: Height-for-age Z score
IYCF: Infant and Young Child Feeding
MAM: Moderate Acute Malnutrition
MICS: Multiple Cluster Surveys
MUAC: Mid-upper Arm Circumference
RUF: Ready-to-use Foods
SAM: Severe Acute Malnutrition
SMART: Standardized Monitoring and Assessment of Relief and Transitions
WA: Weight-for-age
WH: Weight-for-height
WHZ: Weight-for-height Z score
